# A Novel Electrically Small Ground-Penetrating Radar Patch Antenna with a Parasitic Ring for Respiration Detection

**DOI:** 10.3390/s21061930

**Published:** 2021-03-10

**Authors:** Di Shi, Taimur Aftab, Gunnar Gidion, Fatma Sayed, Leonhard M. Reindl

**Affiliations:** Laboratory for Electrical Instrumentation, Department of Microsystems Engineering—IMTEK, University of Freiburg, Georges-Köhler-Allee 106, 79110 Freiburg, Germany; aftab@imtek.uni-freiburg.de (T.A.); gunnar.gidion@imtek.uni-freiburg.de (G.G.); fatma.sayed@imtek.uni-freiburg.de (F.S.); reindl@imtek.uni-freiburg.de (L.M.R.)

**Keywords:** antenna design, ceramic patch antenna, antenna miniaturization, bandwidth improvement techniques, parasitic resonator, ground-penetrating radar, respiration detection

## Abstract

An electrically small patch antenna with a low-cost high-permittivity ceramic substrate material for use in a ground-penetrating radar is proposed in this work. The antenna is based on a commercial ceramic 915 MHz patch antenna with a size of 25 × 25 × 4 mm^3^ and a weight of 12.9 g. The influences of the main geometric parameters on the antenna’s electromagnetic characteristics were comprehensively studied. Three bandwidth improvement techniques were sequentially applied to optimize the antenna: tuning the key geometric parameters, adding cuts on the edges, and adding parasitic radiators. The designed antenna operates at around 1.3 GHz and has more than 40 MHz continuous −3 dB bandwidth. In comparison to the original antenna, the −3 and −6 dB fractional bandwidth is improved by 1.8 times and 4 times, respectively. Two antennas of the proposed design together with a customized radar were installed on an unmanned aerial vehicle (UAV) for a quick search for survivors after earthquakes or gas explosions without exposing the rescue staff to the uncertain dangers of moving on the debris.

## 1. Introduction

The aim of this work is to develop a small-sized and lightweight broadband ground-penetrating radar (GPR) antenna. Generally, a larger antenna can provide resonances in a lower frequency range, enabling better penetration, and has broader bandwidth to achieve more accurate detection—these are, of course, desirable properties for GPR application. However, through the work presented in this article, the authors want to fill the niche of electrically small ground-penetrating radar antennas. Any application that needs a low-cost planar antenna smaller than that of the state of the art with improved bandwidth may find the described antenna design useful. The proposed antenna can be integrated into a small unmanned aerial vehicle (UAV) for searching for and rescuing victims trapped under collapsed buildings after gas explosions or earthquakes by detecting the periodic Doppler shift caused by human chest movements due to respiration. This research was part of the German research project $FOUNT^{2}$ (the German acronym for flying localization system for searching for and rescuing trapped people). In previous work, a bi-quad-antenna was designed [1]. In addition to antenna development, the project also included research on the processing of breathing signals [1,2], a weight-optimized rescue-radar module [3], a weight- and efficiency-optimized multicopter [4], partially autonomous flight control [4], vision-based autonomous landing [5,6], and scenario development for emergency exercises [7]. For lightweight UAV applications, to ensure a certain flight time or to fulfill certain take-off weight regulations, it is essential to reduce the payload weight as much as possible. In our case, the maximum take-off weight of the project UAV is defined as 5 kg. To ensure a flight time of 60 min, the maximum allowed payload weight is 500 g, with a weight budget of 50 g for the two radar antennas.

For the application of the described quick survivor detection radar, the antenna is required to have a low frequency of operation for good penetration through the ground, to have sufficient bandwidth for the acquisition of distance information, and to be lightweight and miniaturized for integration into a UAV.

Metallic objects can reflect almost all of the incident radio-frequency (RF) waves. The interface between two dielectric media can also reflect RF waves due to the impedance mismatch. Based on that, a GPR can detect things underneath a surface. Detecting a human body underneath a collapsed building is a very complex scenario for GPR. A collapsed building consists of many different materials, including wood, brick, concrete, reinforcing steel, and all possible furniture. If some survivors are trapped in it, they may have an air cavity to breath. These different materials and cavities diffusely reflect and attenuate the transmitted RF waves.

The most frequently used antenna type for broadband radar applications is the horn antenna, regardless of if the radar is ultra-wideband (UWB) pulse radar [8,9,10], frequency-modulated continuous-wave (FMCW) radar [11], step-frequency continuous-wave (SFCW) radar [12], or pseudo-noise (PN) radar [13]. Moreover, horn antennas have a very high directivity. Lightweight versions of horn antennas can be found in UAV applications for landmine detection [14,15]. The Vivaldi antenna, as a planar version of the horn antenna, has significantly reduced weight comparing to the classic horn antenna. In recent years, more research has integrated Vivaldi antennas into a UAV for GPR applications, such as landmine detection [16,17]. The maximum radiation of all kinds of horn antennas is directed nearly along the central axis of symmetry to the opening [18]. The opening can be covered with a dielectric material to protect the antenna from the environment. For GPR applications, if the permittivity of the dielectric material and the permittivity of the ground material are the same, the impedance match is optimal. Similarly, the maximum radiation of a Vivaldi antenna is directed along the axis of symmetry. Therefore, for ground penetration, regardless of if the incidence is normal or oblique, only the front edge of the antenna patch touches the ground, which leads to the situation that the main wave first propagates into the air. Most of the radiation is reflected by the air–ground interface.

Another often-used broadband antenna type is the spiral antenna [13,19,20]. In contrast to the horn antenna, the spiral antenna is planar and can touch the ground completely. Miniaturized spiral antennas were studied in [21,22]. The sinuous antenna, as another type of planar broadband antenna, could also be applied as a GPR antenna [23].

If the GPR measures during flight, most of the RF waves transmitted by the antenna cannot penetrate, but will be directly reflected by the ground surface. If the antennas are planar and are mounted under the landing gear of the UAV as feet, when the UAV lands, the antennas can directly touch and couple to the ground without an air gap, so that more RF energy can penetrate the ground to detect the weak chest vibrations of trapped victims. By landing, the UAV motor and devices that are unrelated to the measurement can also be turned off to avoid possible interference caused by the UAV itself.

How can a planar antenna be made small? The most direct way is to use a substrate with high permittivity so that the wavelength is short [24]. An antenna is electrically small when its dimensions are much smaller than the wavelength in the propagation medium. The principle of making an electrically small antenna is lowering the resonance frequency without increasing the dimensions [25]. However, there are some fundamental physical limitations for small antennas. Depending on different criteria, radiation modes, and antenna types, the fundamental limitations are expressed differently [26,27]. The most common way to express the limitations is by deriving the lowest achievable quality factor *Q* because *Q* is the ratio of stored energy to radiated energy. For example, for the lowest-order transverse magnetic (TM) mode mode, the lowest achievable *Q* is

(1) $$Q\approx 1/{\left(kr\right)}^{3},$$

where kr≪1, *k* is the wavenumber, and *r* is the radius of the spherical circumference of the antenna [27]. Within those limitations, the most basic technique for making an antenna electrically small is to improve the space utilization efficiency, as implemented in the fractal antenna [18]. Increasing the number of radiation modes around the operation center frequency or the utilization of metamaterials can also result in electrically small antennas [25].

How can the bandwidth of a planar antenna be increased? Since the quality factor *Q* of an antenna at a resonance frequency $f_{center}$ is reciprocal to the fractional bandwidth FBW [18],

(2) $$Q=\frac{1}{FBW}=\frac{f_{center}}{f_{upper}-f_{lower}}.$$

$f_{upper}$ and $f_{lower}$ are the upper and lower frequency limits of the bandwidth, with $f_{center}$ as the center frequency. Lowering *Q* leads to an increase in the bandwidth. In [28], some general approaches were summarized about how to make a planar broadband antenna: In addition to lowering *Q*, using an impedance-matching network and introducing multiple resonances were suggested.

**Figure 1:**
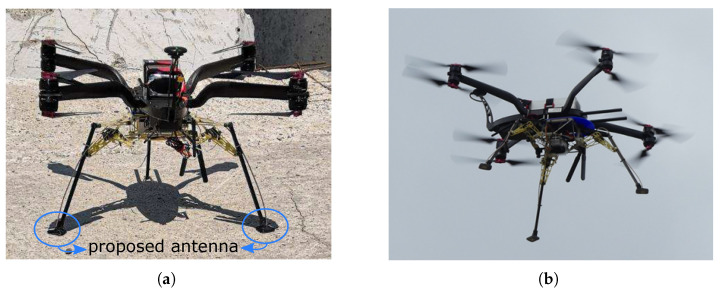
Two of the proposed antennas were integrated into an unmanned aerial vehicle (UAV). (**a**) Landed on the ground; (**b**) during flight.

In this article, an electrically small planar patch antenna is proposed. The authors comprehensively took all of the above-mentioned factors into consideration and applied as many techniques as possible within the restrictions defined by the application. The design procedure in this work was systematically documented and is meant to provide a useful design guideline for small broadband ground-penetrating patch antennas. Two antennas of the proposed design—one for transmitting and one for receiving—were installed as the feet of a UAV and could completely touch the ground when the UAV landed (as in Figure 1), which enabled a direct coupling to the ground. The landing gear of the UAV had three adaptable legs, and each foot could land on a different surface with a minimum area of 2.5 × 2.5 cm^2^ without causing an air gap between the antenna and the ground.

This article is organized as follows. In Section 2, according to the application requirements, the design procedure is defined; the six subsections sequentially introduce the design and optimization methods. Based on the finite element method (FEM), each subsection analyzes the simulated return loss results of the intermediate design. In Section 3, the results of the proposed antenna are presented with its detailed dimensions, eigenmodes, radiation patterns, surface current, electric (E-) field and magnetic (H-) field. The return losses from simulations and measurements are compared. A commercial antenna of a similar frequency range is compared with the proposed antenna. In Section 4, the main work of this article is summarized and the conclusions are given.

## 2. Design Procedure

For good ground penetration, which means less attenuation, the resonance frequency of the antenna should be as low as possible. The radar transmitting power *P* at distance *z* is related to the power $P_{0}$ at the Tx-antenna z=0 by [29]

(3) $$P=P_{0}e^{-2\alpha_{c}z}.$$

The ohmic attenuation coefficient $\alpha_{c}$ is material and frequency dependent. For common ground materials, such as sand (dry, moist, or wet) and clay, the $\alpha_{c}$ increases nonlinearly and monotonically with increasing radar frequency [30].

To estimate a survivor’s range, a bandwidth is needed. The range resolution ΔR is inversely proportional to the radar’s operating bandwidth BW [19]:

(4) $$\Delta R=\frac{c}{2\;\cdot \;BW},$$

where *c* is the electromagnetic wave velocity in a medium. Increasing the bandwidth increases the range resolution. However, for the same substrate material and the same antenna design, simply scaling the antenna dimensions will scale the $f_{center}$ and the BW proportionally, which leaves the fractional bandwidth FBW [29]:

(5) $$FBW=\frac{BW}{f_{center}}$$

which leaves the fractional bandwidth *FBW* unchanged. Therefore, a broader absolute bandwidth BW is easier to achieve if the antenna resonates in a higher frequency range. Over the course of this study, the authors were granted permission to use the RF spectrum from 1.26 to 1.34 GHz by the BNetzA (Federal Network Agency, Bonn, Germany) for research on civil search and rescue. This frequency range provides a compromise between penetration depth and depth resolution.

Considering the requirements of the application on a UAV, the specifications of the antenna are roughly defined as follows:

- The fundamental resonance frequency is around 1.3 GHz;
- Good electromagnetic coupling to common ground/building materials;
- As of a large bandwidth as possible;
- Light weight and small size.

In the following, the guidelines for the design procedure are given in the form of an instruction manual.

### 2.1. Choose an Existing Antenna with a Lower Resonance Frequency

At the beginning of the research, the authors looked for high-permittivity material suppliers for antenna substrates. However, the substrate material parameters are often not stable and the antenna manufacturing for every design iteration is very time consuming.

**Figure 2:**
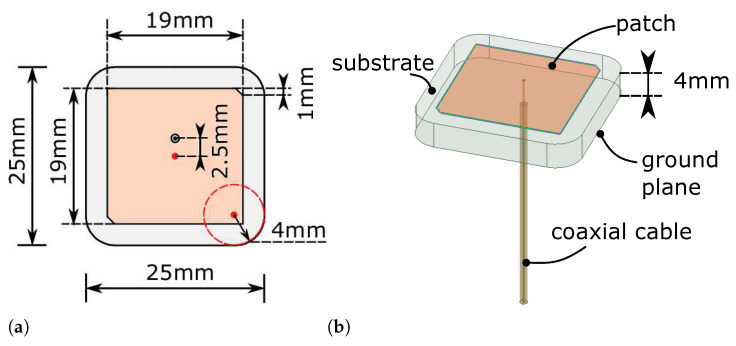
The Abracon 915 MHz antenna. (**a**) Dimensions shown on the top surface; (**b**) trimetric view of the antenna model in the ANSYS High-Frequency Structure Simulator (HFSS).

In this study, the substrate was extracted from a commercially available 915 MHz patch antenna [31]. The antenna is a rectangular cuboid with rounded corners, as can be seen in Figure 2. The size of the rectangular cuboid is 25 × 25 × 4 mm^3^, and the radius of the rounded corners is 4 mm. There is a 19 × 19 mm^2^ silver square patch with two diagonally truncated corners on the front side of the antenna. The back side is fully covered with a silver ground plane. There is a feeding pin located at 2.5 mm from the center point, which goes through the substrate.

The diagonally truncated corners create two orthogonal modes of resonance, making it a classic design of a single-feed circular polarized patch antenna. The earliest publication in which this design was theoretically analyzed is [32]. Later on, based on this basic design, many optimization studies have been reported—for example, by adding cuts on the edges to lower the resonance frequency [33] or by utilizing a U-slot to improve the impedance matching of a thick substrate with the air [34].

The Abracon 915 MHz antenna has a simple design. Within its original patch dimensions, new patterns could be designed and analyzed with the assistance of an RF simulation tool. The extra part could be removed by laser milling. Details about laser milling are described in Section 2.6. The simulation environment that was used in this work was the ANSYS High-Frequency Structure Simulator (HFSS). All of the simulations in this work used the discrete frequency sweep type for more accurate results.

### 2.2. Extract the Substrate Permittivity Using an Iterative FEM Simulation

For further simulations, it is necessary to have all of the important material parameters of the substrate. As the substrate was designed to be used as antenna substrate, we assumed that the relative magnetic permeability is 1, the conductivity is 0, and the dielectric loss tangent is 0. The only important parameter remaining unknown is the relative permittivity $\epsilon_{r}$.

**Figure 3:**
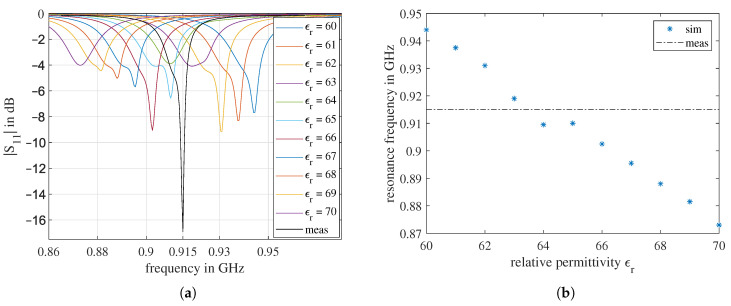
Measurement of the Abracon 915 MHz antenna and results of the sweep $\epsilon_{r}$ simulation: (**a**) the reflection coefficient $|S_{11}|$ over frequency; (**b**) the influence of $\epsilon_{r}$ on the antenna resonance frequency $f_{r}$. The measured resonance frequency is highlighted with a horizontal dashed line.

The center frequency given in the data sheet is 915 MHz, which was also confirmed by the network analyzer measurement: The black curve in Figure 3a shows the magnitude of the $S_{11}$ coefficient of the unmodified antenna.

The propagation speed of electromagnetic waves in a medium depends on the relative permittivity $\epsilon_{r}$ and the relative permeability $\mu_{r}$. Since antenna substrates are dielectric materials, the $\mu_{r}$ is always one. The relative permittivity $\epsilon_{r}$ of the substrate at $f_{r}=$ 915 MHz can be estimated using the equation:

(6) $$f_{r}\approx \frac{c_{0}}{\sqrt{\epsilon_{r}}\;\cdot \;\frac{L}{2}},$$

where *L* is the longest possible current path on the patch surface, which is approximately the circumference of the patch. For the original patch L≈4× 20 mm = 80 mm, so that

(7) $$\epsilon_{r}\approx 4\;\cdot \;{\left(\frac{c_{0}}{L\;\cdot \;f_{r}}\right)}^{2}\approx 67\pm 10\%.$$

The exact antenna model in Figure 2 was established in the HFSS. The $\epsilon_{r}$ was set as a project variable and swept from 60 to 70. The $S_{11}$ curves obtained from the simulation are shown in Figure 3a. The minima of the $S_{11}$ curves are the resonance frequencies $f_{r}$ in Figure 3b. It is easy to conclude from the results that, with increasing $\epsilon_{r}$, the antenna resonance frequency decreases. The only exception happens at $\epsilon_{r}=65$. However, in comparison with Figure 3a, it can be seen that from $\epsilon_{r}=64$ to $\epsilon_{r}=65$, a new resonance appears, but the center of the resonating frequency band continuously moves to the left, which matches the tendency.

According to Figure 3, the $\epsilon_{r}$ of the antenna substrate at 915 MHz should be between 63 and 64. However, due to dispersion, the $\epsilon_{r}$ at 1.3 GHz has a different value. Since the dielectric dispersion function of this substrate material is unknown, for each simulation, a constant value is used. Therefore, a short-term simulation, fabrication, and measurement in a loop is essential to extract more accurate material parameters. After several iterations, it was observed that simulations with $\epsilon_{r}=62$ match the measurement around 1.3 GHz best; therefore, we used $\epsilon_{r}=62$ for further simulations. The intermediate results of the simulation and measurement iterations to extract this value are tedious and not relevant to the main part of this work; thus, the description of this part is omitted.

### 2.3. Estimate the Necessary Patch Dimensions for Air-Coupled Scenarios

In order to increase the resonance frequency from 930 MHz to 1.3 GHz, the patch size should be reduced. From some initial simulations, we found out that in addition to the patch width patchW, the patch center position *xOffset* and the width of the diagonal corners’ truncation cornerCutW also have dramatic an impact on the antenna’s resonance frequency $f_{r}$.

**Figure 4:**
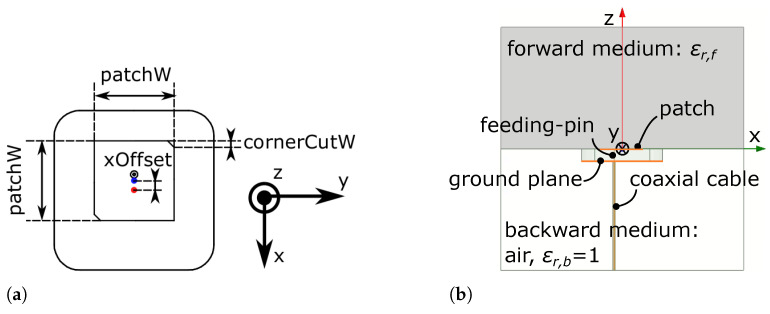
(**a**) The three key dimension parameters on the antenna’s top surface: patchW: the width of the square patch, *xOffset*: the patch center in relation to the substrate center, cornerCutW: the side length of the diagonally truncated corners. (**b**) Side view of the HFSS simulation antenna model. The antenna is surrounded by a 75×75× 75 mm^3^ box. The lower half of the box is air. The material of the upper half can be changed according to the simulation scenario.

Thus, we chose these three as key dimension parameters for simulation and analysis, as illustrated in Figure 4a. It should be emphasized here that, because the pin is built into the substrate, by changing the *xOffset*, we are changing the position of the feeding pin relative to the patch center. The *xOffset* of the original antenna is 0.

The side view of the antenna simulation model is shown in Figure 4b. The antenna is surrounded by a 75×75× 75 mm^3^ box. The surface of this box is defined as a radiation boundary. The antenna patch is on the upper surface of the substrate. Therefore, the upper half of the box is the forward wave propagation medium with a relative permittivity of $\epsilon_{r,f}$. The lower half of the box is the backward wave propagation medium, which is air, so the $\epsilon_{r,b}$ is always 1. In an air-coupled scenario, $\epsilon_{r,f}=1$. In the ground-penetrating scenario, depending on the ground material, the $\epsilon_{r,f}$ is different and is always larger than 1.

**Figure 5:**
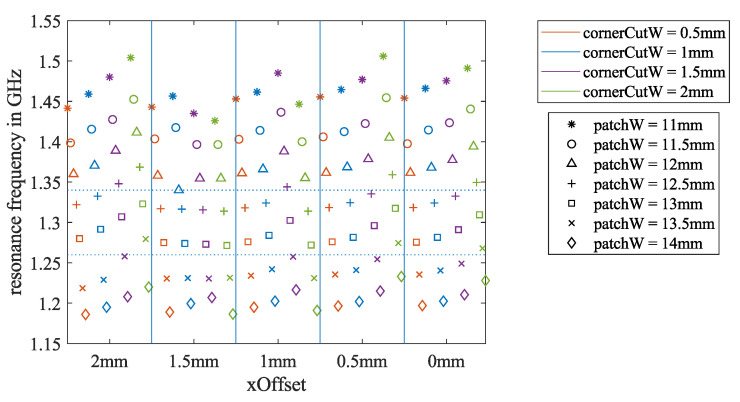
Results of all 140 simulation iterations to estimate the required patch dimensions for the air-coupled antenna scenario. They show the dependency of the resonance frequency $f_{r}$ on the three key dimension parameters.

In the air-coupled simulation, the patchW was swept from 11 to 14 mm, the *xOffset* was swept from 2 to 0 mm, and the the cornerCutW was swept from 0.5 to 2 mm. All three parameters were changed with a step size of 0.5 mm. In total, there were 7×5×4=140 iterations in this simulation. The minimum peak of the calculated $S_{11}$ curve was defined as the resonance frequency $f_{r}$. The $f_{r}$ values of all 140 iterations are summarized in Figure 5. In five separate boxes, corresponding to five different *xOffset* values, the resonance frequency is plotted against the four-color-coded cornerCutW values. Using seven different symbols, the impact of patchW is also shown.

As expected, for the same *xOffset* and the same cornerCutW, the resonance frequency $f_{r}$ decreases when the patchW increases. Additionally, in most cases, for the same patchW, when the cornerCutW rises, the $f_{r}$ also rises. However, no conclusion about the impact of *xOffset* on $f_{r}$ could be drawn from Figure 5.

The two horizontal dotted lines in Figure 5 mark the frequency band between 1.26 and 1.34 GHz that we were permitted to use for this project. The $f_{r}$ values of the simulations with patchW = 12.5 and 13 mm were mainly in this frequency range. Hence, we focused on more simulations for patch widths in this range.

### 2.4. Estimate the Necessary Patch Dimensions for Ground-Coupled Scenarios

**Figure 6:**
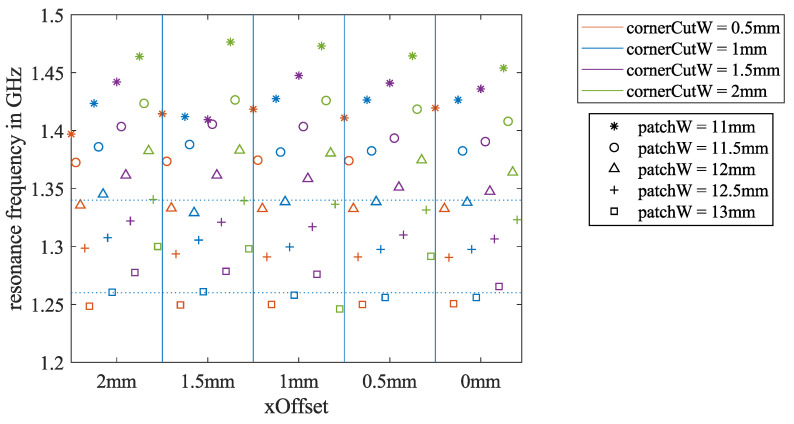
Results of all 100 simulation iterations for estimating the required patch dimensions for the ground-coupled antenna scenario. The simulated ground material has $\epsilon_{r,f}=4$. This shows the dependency of the resonance frequency $f_{r}$ on the three key dimension parameters.

**Figure A1:**
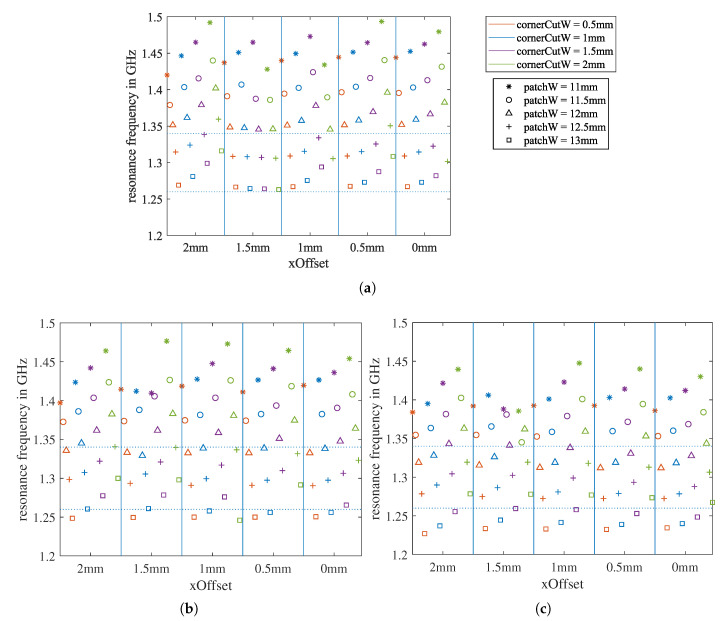
Simulation results with a sweep of the three key geometric parameters for the ground-coupled antenna scenario described in Section 2.4. The relative permittivity of ground material is: (**a**) $\epsilon_{r,f}=2$; (**b**) $\epsilon_{r,f}=4$; (**c**) $\epsilon_{r,f}=6$.

From the literature, it can be found that the relative permittivity of common building materials, such as concrete and bricks, depends on age and RF. For the frequency range that we are working in, the relative permittivity of those materials varies from 2 to 9 [30,35,36,37]. To simplify the problem, we assumed that the $\epsilon_{r,f}$ of the ground material is constant in the frequency range of interest and repeated the simulations from Section 2.3 for $\epsilon_{r,f}=2,4$, and 6, respectively. A summary of the resonance frequency depending on the three key dimension parameters for $\epsilon_{r,f}=4$ is shown in Figure 6. The results of $\epsilon_{r,f}=2$ and 6 can be found in Appendix A, Figure A1. Compared to Figure 5, the resonance frequencies of all the iterations were lower when the antenna was touching the ground. For the ground material of $\epsilon_{r,f}=4$, the results of patchW= 12.5 mm exhibited resonance frequencies closest to 1.3 GHz.

### 2.5. Increase the Bandwidth

There are many techniques for increasing the antenna bandwidth. We researched the following techniques.

#### 2.5.1. Bandwidth Extension by Tuning the Geometric Parameters and Feeding Point Position

**Figure 7:**
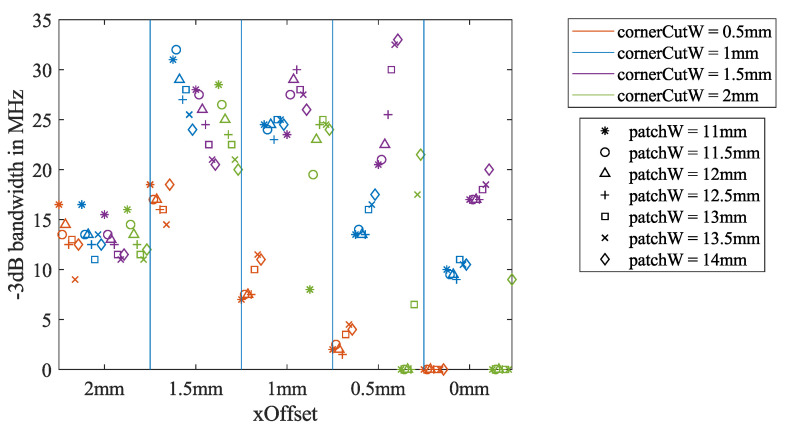
Dependency of the return loss for the −3 dB bandwidth on the three key dimension parameters, calculated from the air-coupled simulation described in Section 2.3.

**Figure 8:**
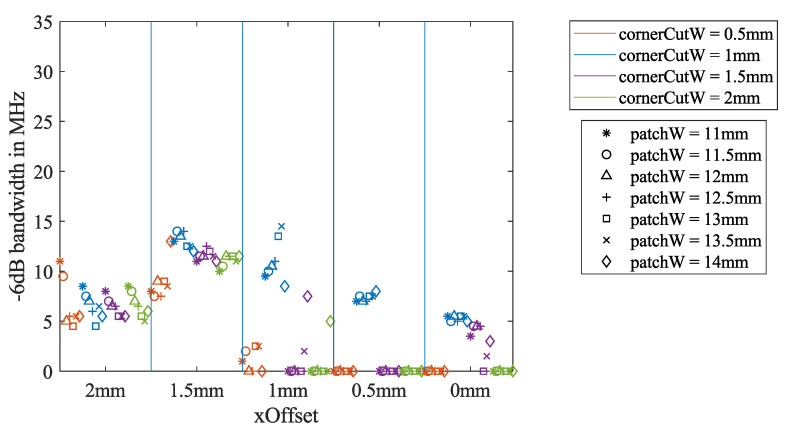
Dependency of the −6 dB bandwidth on the three key dimension parameters, calculated from the air-coupled simulation described in Section 2.3.

From the simulation in Section 2.3, the influence of the three key dimension parameters on the return loss bandwidth could be analyzed as well. The −3 dB bandwidth is summarized in Figure 7, and the −6 dB bandwidth is summarized in Figure 8. For *xOffset* = 2 and 1.5 mm, the −3 dB bandwidth is reduced when the patchW increases. For *xOffset* = 0.5 mm, the relationship is the opposite. Other dependencies of the bandwidth on the three parameters are hard to identify.

For the −6 dB bandwidth, the inverse proportionality to patchW can hardly be observed from the *xOffset*= 2 mm section. For *xOffset* = 1, 0.5, and 0 mm, many iterations have no −6 dB bandwidth. However, all of the blue markers for cornerCutW= 1 mm have the −6 dB bandwidth in all of the *xOffset* sections. In addition, the blue markers have the largest −6 dB bandwidth for *xOffset* = 1.5 and 1 mm.

**Figure A2:**
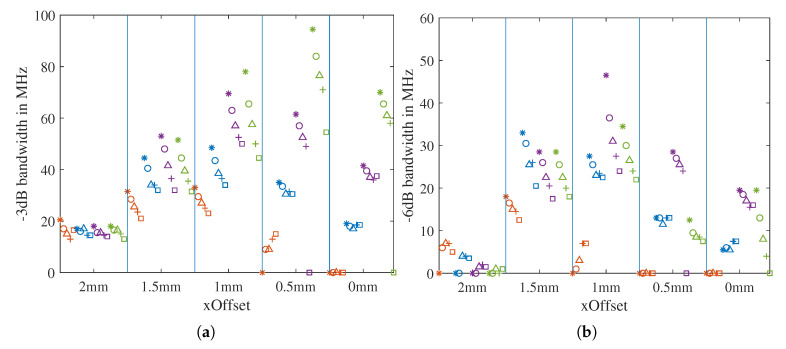
The bandwidth of the simulation described in Section 2.5.1 calculated for ground material with $\epsilon_{r,f}=4$: (**a**) −3 dB bandwidth; (**b**) −6 dB bandwidth.

The dependency of the return loss bandwidth on the three key parameters for the ground-coupled scenario with $\epsilon_{r,f}=4$ can be found in Appendix A, Figure A2. As shown in Figure A2b, for $\epsilon_{r,f}=4$, the iterations with cornerCutW= 1 mm also exhibit a relatively broad −6 dB bandwidth for *xOffset* = 1.5 mm. For *xOffset* = 1 mm, the iterations of cornerCutW= 1.5 and 2 mm present wider bandwidths than those of cornerCutW= 1 mm, but for patchW = 12.5 mm, with which the resonance frequency is closest to 1.3 GHz, the difference is very small. Thus, we define patchW = 12.5 mm and cornerCutW= 1 mm and choose *xOffset* = 1.5 and 1 mm for further comparison.

**Figure 9:**
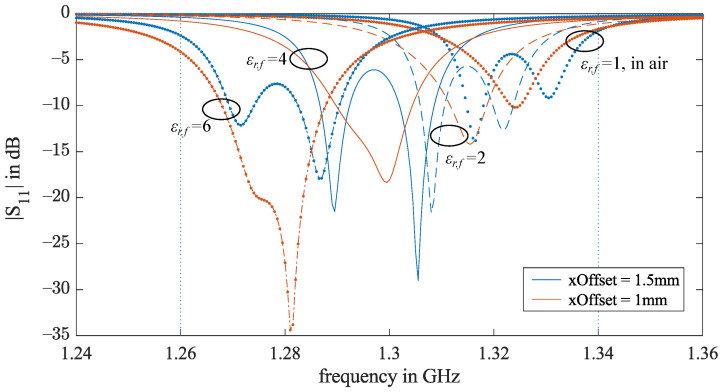
patchW = 12.5 mm and cornerCutW= 1 mm. The return losses of *xOffset* = 1.5 and 1 mm from the air-coupled and ground-coupled simulations. The relative permittivity $\epsilon_{r,f}$ of the the ground material is 2, 4, or 6.

In Figure 9, eight return loss curves from the simulations described in Section 2.3 and Section 2.4 are presented. The results for *xOffset* = 1.5 mm are in blue and those for 1 mm are in red. It is common to both cases that, when the ground material $\epsilon_{r,f}$ increases, the antenna resonance is enhanced and the resonance frequency decreases. Even though the bandwidth of *xOffset* = 1.5 mm is always wider, the two resonances are far from each other in comparison to the *xOffset* = 1 mm iterations. For *xOffset* = 1 mm, the two resonances are close enough and sufficient to produce one enhanced continuous bandwidth. A continuous bandwidth allows the radar to be operated as an SFCW radar with a flexible frequency step choice. Therefore, we choose *xOffset* = 1 mm for further optimization.

#### 2.5.2. Bandwidth Extension Using Cuts on the Patch Edges

Adding small cuts on the patch edges could increase the current path length and, thus, decrease the fundamental resonance frequency. This is the same principle as that for the fractal antennas [18]. The cuts may also introduce some neighboring resonances of the antenna; therefore, we systematically add cuts on the antenna patch edges and analyze if the bandwidth improves.

**Figure 10:**
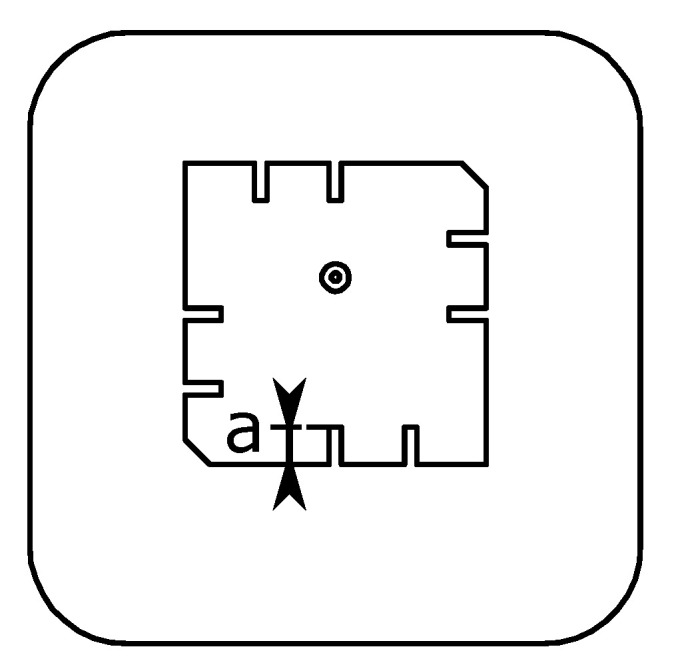
There are two cuts on each edge of the patch. The length of the cuts is the variable *a*. In this simulation, patchW= 12.5 mm, *xOffset* = 1 mm, and cornerCutW= 1 mm.

As shown in Figure 10, we add two cuts on each edge. For the upper edge, one cut is located in the middle, and the second one is in the middle of the left half of the edge. The cuts are distributed symmetrically around the patch center. The idea of this design is to keep the circular polarization property of the original design. The width of the cuts is a constant value of 0.5 mm; their length is the variable *a* and varies from 0 to 3 mm.

**Figure 11:**
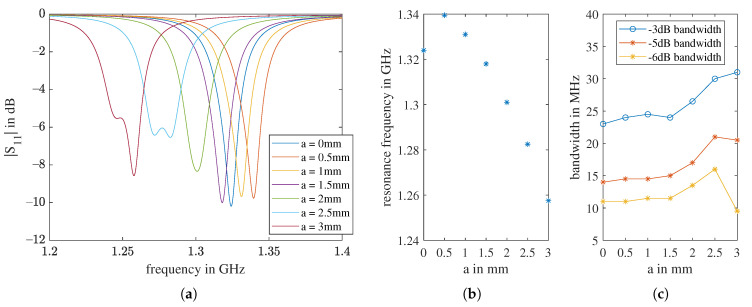
The simulation results of changing the length of the cuts *a* for the air-coupled scenario: (**a**) reflection coefficient $S_{11}$ curves of all values of *a*; (**b**) the influence of *a* on the antenna resonance frequency $f_{r}$; (**c**) the influence of *a* on the antenna bandwidth $f_{r}$.

The simulation results can be seen in Figure 11. From the patch with no cuts to the patch with the smallest cuts of a= 0.5 mm, the resonance jumps firstly to a higher frequency, then decreases with increasing *a*. The bandwidth remains almost the same until a= 1.5 mm, then starts to grow, and reaches its maximum −6 dB bandwidth by a= 2.5 mm. Here, the two basic resonances are a slightly apart from each other and almost equally strong. At a= 3 mm, the first resonance is weakened, and the −6 dB bandwidth drops. We choose a= 2 mm to proceed because, for this value, the two resonances are still very close to each other and the useful bandwidth is centered closest to 1.3 GHz.

#### 2.5.3. Bandwidth Extension Using a Parasitic Radiator

The bandwidth could be enlarged by adding some parasitic radiators adjacent to the driving element. In [38], the additional passive radiators were rectangular patches, which had a similar size to that of the active one in the center. It was shown in [39] that the bandwidth could be broader even when the parasitic elements were gap-coupled to the non-radiating edges of the driving patch. In [40], the active patch was round, and the parasitic one had a ring shape and encircled the active patch. In [41], the active patch had an elliptical ring shape and encircled the passive patch. In all of these designs, the parasitic patches were very close to the active patch, and they coupled electromagnetically to the active one through a small gap. In most cases, the parasitic elements had a similar size to that of the driving patch, which means that their resonances were close to each other, but not the same. If all adjacent resonances are close enough to combine, a wider bandwidth can be achieved.

**Figure 12:**
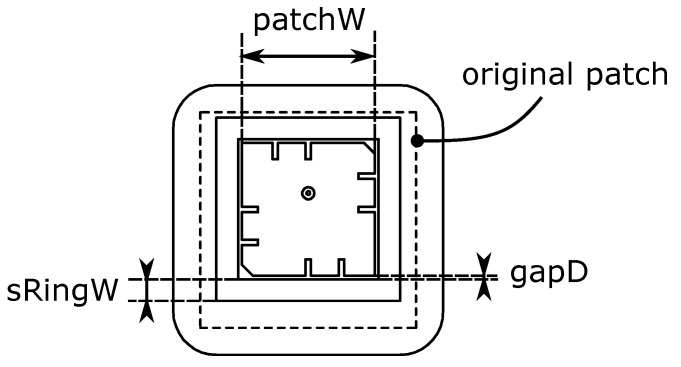
A square-shaped ring is added around the patch. In this simulation, a= 2 mm, *xOffset* = 1 mm, and cornerCutW= 1 mm.

Within the design constraints, a square-shaped ring was selected as the parasitic radiator for this design study. As shown in Figure 12, two new geometric parameters are defined: sRingW describes the width of the ring and gapD describes the width of the gap between the original patch and the parasitic ring.

**Figure 13:**
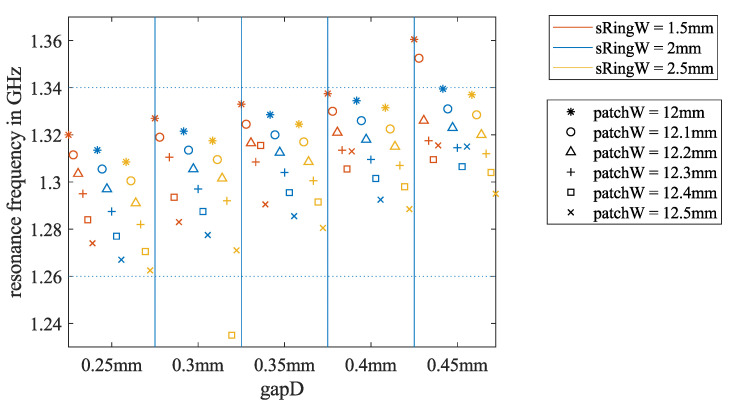
Resonance frequencies from the air-coupled parametric simulation for the design with a square-shaped parasitic ring. The sRingW sweeps from 1.5 mm with a step size of 0.5 to 2 mm. The gapD sweeps from 0.25 mm with a step size of 0.05 to 0.45 mm. In the meantime, the patchW finely changes from 12 to 12.5 mm with a step of 0.1 mm.

**Figure 14:**
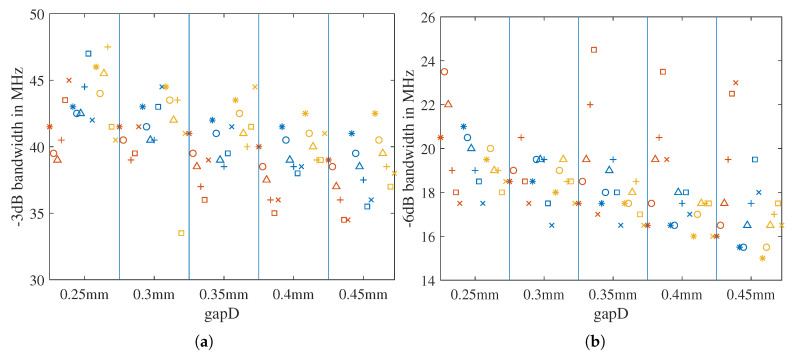
Bandwidth as a function of the three key dimension parameters, sRingW, gGap, and patchW, for the design with a square-shaped parasitic ring: (**a**) −3 dB bandwidth; (**b**) −6 dB bandwidth.

The parametric simulation results can be seen in Figure 13 and Figure 14. From the results, we come to the following observations: With increasing gap width gapD, the resonance frequency $f_{r}$ increases, and the bandwidth mainly decreases. With a thicker ring width sRingW, the $f_{r}$ drops and the −3 dB bandwidth increases, but the −6 dB bandwidth decreases. Considering the advantages and disadvantages for the resonance frequency position and the bandwidth value, we choose gapD= 0.35 mm, sRingW= 2 mm, and patchW= 12.3 mm for the final design.

**Figure 15:**
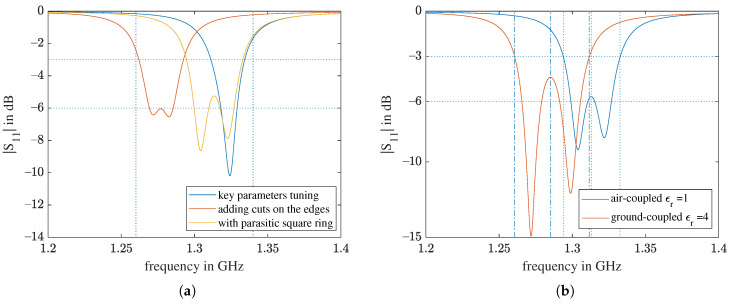
Main numerical results of the bandwidth improvement: (**a**) comparison between results of the three subsequently applied bandwidth-increasing techniques for *xOffset* = 1 mm and cornerCutW= 1 mm. The patchW of the first two designs is 12.5 mm; that of the third one is 12.3 mm. (**b**) Comparison between the air-coupled simulation and the ground-coupled simulation with $\epsilon_{r,f}=4$ for the final design.

The main results of applying the three bandwidth improvement techniques are summarized in Figure 15a, and the exact values are in Table 1. For the final design, the simulation was repeated for touching a material box with $\epsilon_{r,f}=4$. As shown in Figure 15b, the radiating bandwidth of the ground-coupled simulation was shifted to the left in comparison to the air-coupled simulation, as expected. However, for the final design, the −3 dB bandwidths of both situations were within the granted 1.26–1.34 GHz band. The bandwidths of the final and original designs are summarized in Table 2.

### 2.6. Optimization with a Short-Term Simulation, Fabrication, and Measurement in a Loop

As mentioned in Section 2.2, due to dielectric dispersion, the relative permittivity of the substrate at the specified 1.3 GHz differs from the permittivity at the working frequency 915 MHz of the original antenna. Using a short-term loop of simulation, fabrication, and measurement, almost-realistic material parameters can be derived, and a foundation for more accurate simulations can be set. Moreover, fabrication at an early stage will help researchers learn the limits of accuracy in the fabrication. Delicate simulation steps beyond the accuracy of fabrication cannot be implemented and verified.

**Figure 16:**
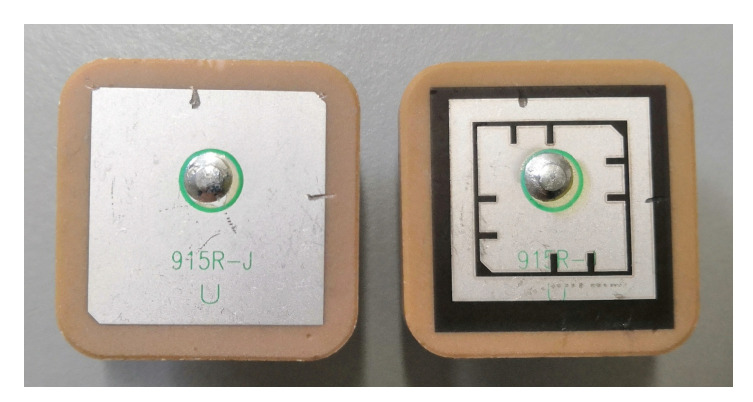
Antenna before and after.

One possibility to quickly and accurately modify an existing antenna is by using laser milling. Our institute is equipped with an ultraviolet pulse laser marking machine from Trumpf: a TruMark Station 5000 combined with a TruMark 6330 laser. On the part of the antenna patch that was irradiated by the pulse laser, the silver was oxidized, turned black, and lost its conductivity, as can be seen in the specimen on the right-hand side in Figure 16. The main parameters of the applied laser milling process are listed in Table 3.

## 3. Results and Discussion

### 3.1. Proposed Antenna

The parameters of the proposed antenna are summarized in Table 4. On the on the left-hand side of Figure 16 is the original commercial antenna from Abracon [31]; on the right-hand side is the proposed antenna that was modified by the above-mentioned laser-marking machine.

### 3.2. Eigenmodes

Eigenmode-analysis simulations were conducted for both the air-coupled and ground-coupled ($\epsilon_{r,f}=4$) scenarios. Table 5 lists the first eight modes from the simulation. The minimum frequency for analysis was 100 MHz. For both scenarios, the first mode was the same: 249 MHz, which is the eigenmode of the coaxial cable. The second to fourth modes were caused by structure combinations, such as the substrate, the ground plane, and the cable. The fifth and sixth modes were the two fundamental eigenmodes of the antenna patch with the parasitic element; they are the focus of this work.

The *Q* factors of modes 5 and 6 were calculated with Equation (2). $f_{upper}$ and $f_{lower}$ are the frequencies marked by the vertical lines in Figure 15b, at which the return loss was equal to −3 dB or the local maximum near the corresponding eigenmode frequency $f_{center}$. For both scenarios, the two modes had a similar *Q* factor.

### 3.3. Radiation Patterns

**Figure 17:**
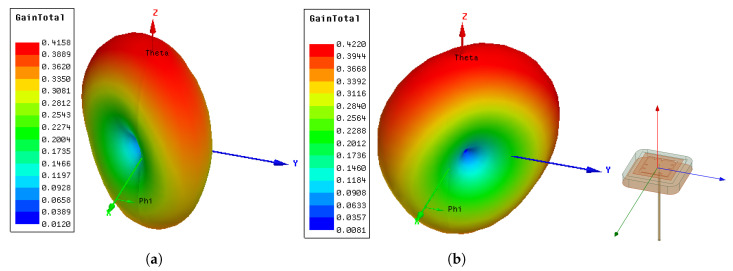
Radiation patterns of the final design from the ground-coupled ($\epsilon_{r}=4$) simulation at: (**a**) 1.272 GHz; (**b**) 1.299 GHz.

The radiation patterns of the final designed antenna at modes 5 and 6 from the ground-coupled ($\epsilon_{r,f}=4$) simulation are shown in Figure 17. The radiation patterns of both modes had similar donut shapes and were approximately perpendicular to each other in the xy-plane. The *z*-direction forward lobe was larger than the back lobe in both modes.

### 3.4. Surface Current, E-Field, and H-Field

**Figure 18:**
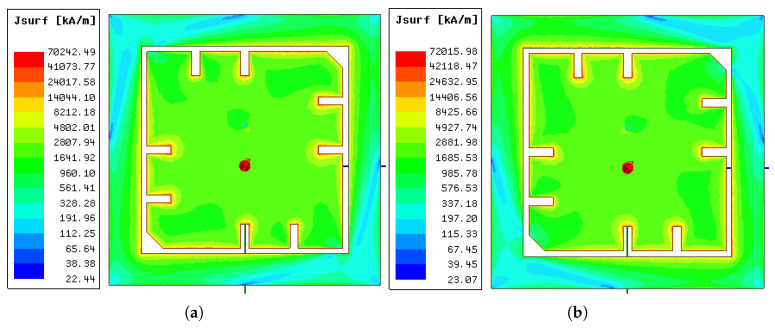
Surface current distribution $J_{xy}$ at $phase=90^{\circ }$ from the ground-coupled ($\epsilon_{r,f}=4$) simulation in: (**a**) mode 5; (**b**) mode 6.

The surface current density $J_{surf,xy}$ at $phase=90^{\circ }$ of the two fundamental eigenmodes can be seen in Figure 18. Corresponding to the radiation patterns, the main current paths of the two modes were also perpendicular to each other. The surface current of the lower mode took the diagonal path on which the main patch’s corners were truncated. In both modes, the square-shaped parasitic ring was well excited.

**Figure 19:**
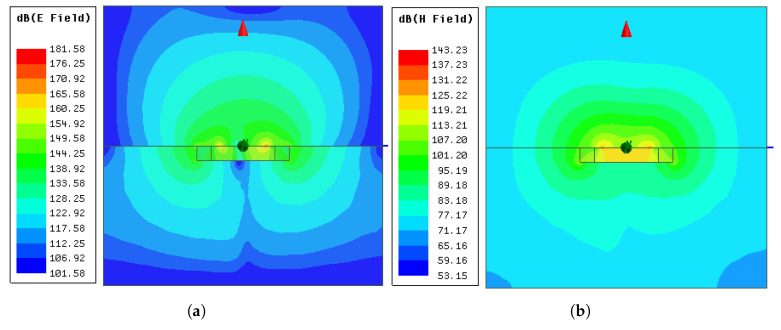
Electric and magnetic fields of mode 5 from the ground-coupled ($\epsilon_{r}=4$) simulation: (**a**) $E_{yz}$ at $phase=0^{\circ }$; (**b**) $H_{yz}$ at $phase=90^{\circ }$.

**Figure 20:**
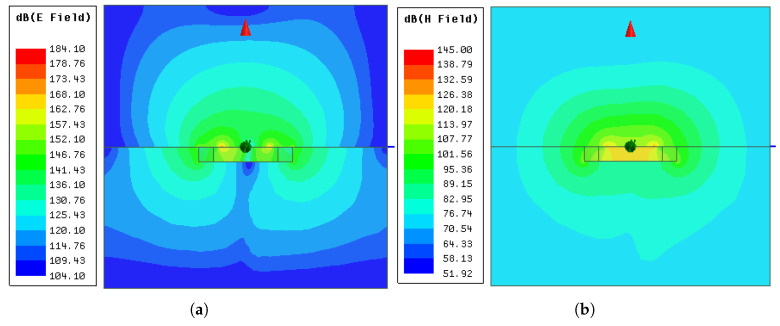
Electric and magnetic fields of mode 6 from the ground-coupled ($\epsilon_{r}=4$) simulation: (**a**) $E_{yz}$ at $phase=0^{\circ }$; (**b**) $H_{yz}$ at $phase=90^{\circ }$.

The electric and magnetic fields of modes 5 and 6 from the ground-coupled ($\epsilon_{r,f}=4$) simulation in the yz-plane are presented in Figure 19 and Figure 20. It can be seen that the fields generated by the antenna can properly penetrate a material with $\epsilon_{r,f}=4$. As expected, the H-field was continuous at the interface and the E-field lines had discontinuities due to the change in permittivity.

### 3.5. Return Loss

**Figure 21:**
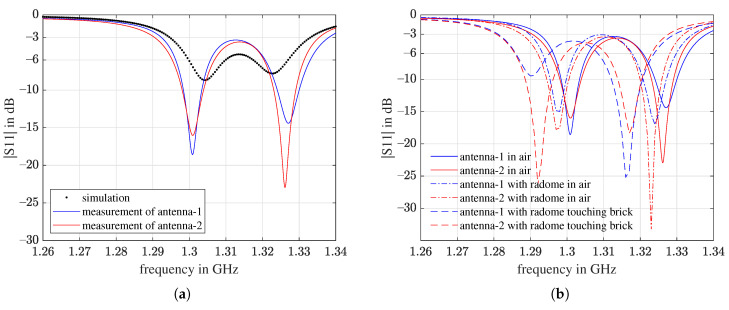
Return loss measurements of two antennas of the proposed design. (**a**) In air, without the silicone rubber radome, including the air-coupled simulation; (**b**) in air, with and without the silicone rubber radome, and touching brick with the silicone rubber radome.

Two pieces of the final design antenna were fabricated and a series of return loss measurements were conducted. The measured return loss in Figure 21a shows that the performance of the two antennas is very similar. In comparison to the simulation results in the same plot, the two resonances of the actual antennas were deeper and further away from each other. One of the reasons is that the substrate material is dispersive, but we assigned a constant $\epsilon_{r}$ of 62 for the entire frequency range in the simulation.

**Figure 22:**
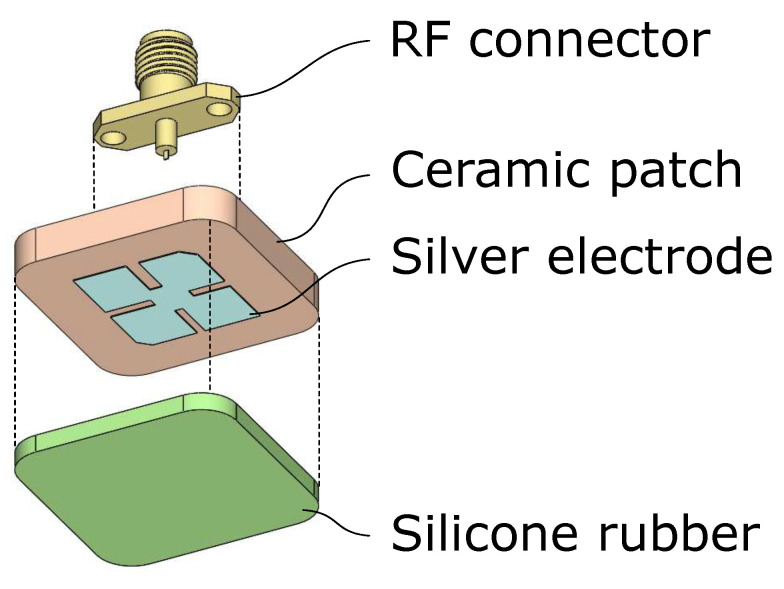
Antenna assembly with a silicone rubber radome.

To protect the fragile ceramic patch from shattering when the UAV has a hard landing on the ground, a silicone rubber layer was added on top of the antenna as a radome, as illustrated in Figure 22. The measurement results in Figure 21b show that with the silicone rubber layer, the return loss was shifted to the left by about 30 MHz. If the antenna touches a brick, Figure 21b, the curves shift further to the left. Compared with the $S_{11}$ spectra from the simulations in Figure 15, it can be inferred that the relative permittivity of the brick we used for the measurements is less than 4.

From the measurements with the silicone rubber radome, the average −3 dB bandwidths for the air-coupled and ground-coupled scenarios are 46.5 MHz and 48 Hz, respectively, and the average −6 dB bandwidths for the air-coupled and ground-coupled scenarios are 22 MHz and 26 Hz, respectively.

Assuming that the radar operates with a bandwidth of 40 MHz, according to Equation (4), the range resolutions in air and in ground material with $\epsilon_{r,f}=4$ are 3.75 and 1.875 m, respectively. In a real scenario, the ground material is unknown and is usually a mixture of different kinds, so the resolution calculation here is only an estimation. With this order of resolution, it is impossible to distinguish between two trapped survivors if they are close. However, it is sufficient to eliminate interference or ghost signals beyond a certain distance.

### 3.6. Comparison with a Commercial Antenna

**Figure 23:**
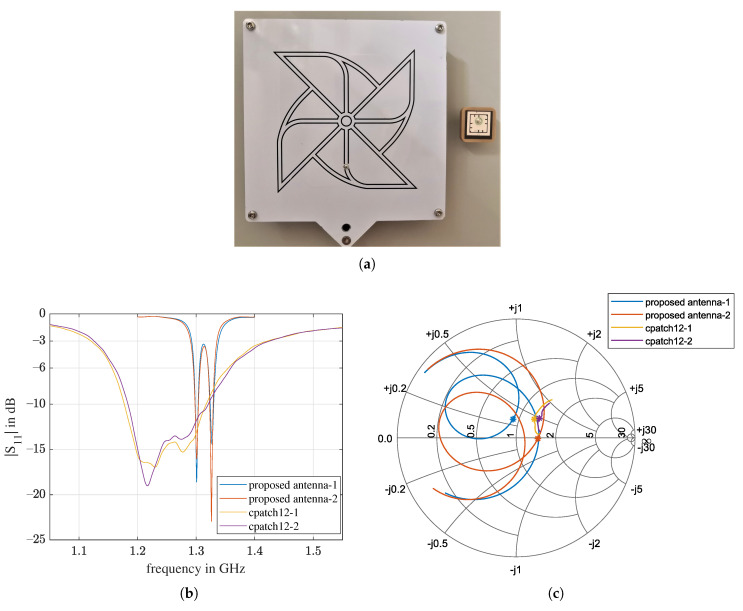
Comparison of the proposed antenna with a commercial circular patch antenna, cpatch12. (**a**) On the left is the commercial antenna [42]; on the right is the proposed antenna; (**b**) measured return losses of two antennas of the proposed design and two cpatch12 antennas; (**c**) Smith chart of the measurement in (**b**). The frequency ranges for all four curves are from 1.26 to 1.34 GHz. The impedance at 1.3 GHz is marked with ∗.

Here, we compare a commercial circular polarized antenna, cpatch12 [42], with a center frequency of 1.265 GHz, to the proposed antenna. Figure 23a is a photo of the two antennas. The measured return loss in Figure 23b shows that the cpatch12 antenna has a much broader bandwidth. The Smith chart in Figure 23c shows that the impedance of cpatch12 antenna is better matched to 50 Ohm. However, the proposed antenna exhibits resonances in the same frequency range as the compared commercial one, even though its volume is reduced by over 100 times. Moreover, the surface of the cpatch12 antenna is 169 cm^2^, which is 27 times as large as the surface of the proposed antenna. The larger surface of the antenna requires a larger flat area on the rubble to avoid air gaps, which lead to poor antenna–ground coupling. In a real disaster scene, it can be very difficult for the UAV to find a suitable landing site to begin with.

The measured specifications are further summarized in Table 6. The volume and the weight of the proposed antenna were only 0.8% and 9.2% of those of the cpatch12 antenna. The −3 dB fractional bandwidth FBW was 14.7%, which was greater than 0.8% and 9.2%, which means that the miniaturization does not have as much of an impact on the FBW as it does on the volume and weight.

## 4. Conclusions

In this work, a miniaturized ground-penetrating patch antenna was designed and fabricated. The antenna design is based on a low-cost 2.5 cm^3^ commercial ceramic patch antenna. By using laser milling, the size of the existing patch could be reduced to the proposed design. The design was systematically conducted. The design guidelines are summarized as follows:

1. Starting point: An existing patch antenna that has a lower resonance frequency than the desired specification;
2. Extract the relative permittivity of the substrate at the desired operating frequency through RF simulation and verify with measurements;
3. Use the estimated permittivity as a material parameter and estimate the dimensions needed for an air-coupled scenario by applying parametric sweep simulations of the key geometric parameters;
4. For ground-penetrating applications, change the relative permittivity of the forward wave propagation medium and repeat step 3.
5. Apply bandwidth improvement techniques:
  - Tune the key geometric parameters: patch width, feeding point position, and corner truncation size;
  - Add cuts on the edges;
  - Add parasitic elements.
6. Optimization with a short-term loop of simulation, fabrication, and measurement.

The proposed antenna was designed for use in a ground-penetrating radar system that radiates into common building materials. The working frequency range is between 1.26 and 1.34 GHz. For the air-coupled case, the fractional bandwidth for the −6 dB return loss of the proposed antenna is 1.5%, which is an improvement of four times with respect to the original antenna. The experiment described in Appendix B shows that the proposed antenna is suitable for use as an RF antenna for ground-penetrating respiration detection. 

## Figures and Tables

**Table 1:** Bandwidth improvement with the analyzed techniques.

After	Key Parameter Tuning	Adding Cuts on the Edges	Adding Parasitic Square Ring
**−3 dB Bandwidth**	23 MHz	30 MHz	38.5 MHz
**−6 dB Bandwidth**	11 MHz	16 MHz	19.5 MHz

**Table 2:** Bandwidth improvement between the original and proposed antennas.

	Center Frequency	−3 dB Bandwidth	Percentage	−6 dB Bandwidth	Percentage
**original antenna**	915 MHz	15 MHz	1.6%	3.5 MHz	0.4%
**proposed antenna**	1.3 GHz	38.5 MHz	3.0%	19.5 MHz	1.5%
**improvement**			1.8 times		4 times

**Table 3:** Main parameters of the applied laser milling process.

Mode	Pulse Frequency	Track Width	Power	Velocity
pulsed	20 kHz	0.015 mm	100%	200 mm/s

**Table 4:** Parameters of the final design.

Patch Width patchW	Offset from Center *xOffset*	Corner Truncation Size cornerCutW	Length of Cuts *a*	Width of Parasitic Ring sRingW	Gap Width gapD
12.3 mm	1 mm	1 mm	2 mm	2 mm	0.35 mm

**Table 5:** Eigenmode simulation results.

	Air-Coupled		Ground-Coupled ($\epsilon_{r,f}=4$)	
**Eigenmode**	**Frequency**	Q	**Frequency**	Q
**mode 1**	249 MHz		249 MHz	
**mode 2**	772 MHz		504 MHz	
**mode 3**	842 MHz		832 MHz	
**mode 4**	850 MHz		839 MHz	
**mode 5**	1.291 GHz	68	1.270 GHz	52
**mode 6**	1.320 GHz	68	1.296 GHz	49
**mode 7**	1.482 GHz		1.469 GHz	
**mode 8**	1.710 GHz		1.571 GHz	

**Table 6:** Comparison between the proposed antenna and a commercial antenna, cpatch12.

	Center Frequency	−3 dB Bandwidth	FBW	Dimension	Weight
**proposed antenna**	1.3 GHz	45.4 MHz	0.035	2.5×2.5×0.4=2.5 cm^3^	12.9 g
**cpatch12**	1.265 GHz	300 MHz	0.237	13×13×1.8=304.2 cm^3^	140 g
**comparison**			14.7%	0.8%	9.2%

**Figure A3:**
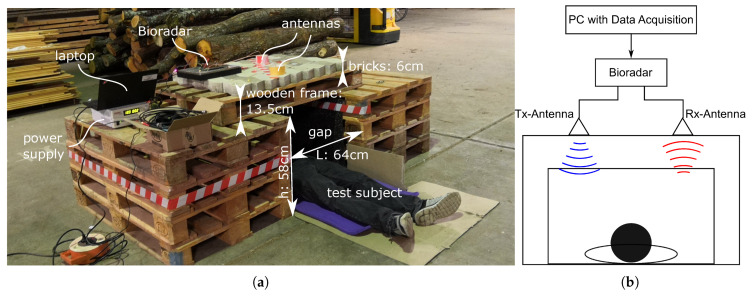
Experimental setup. (**a**) Picture. (**b**) Schematic illustration.

**Figure A4:**
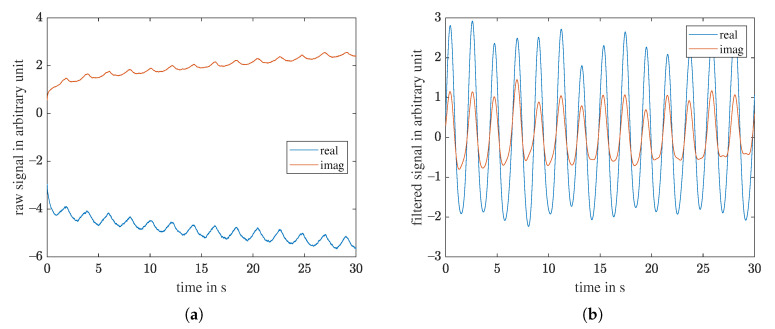
Measured breathing signal from the experiment illustrated in Figure A3. (**a**) Raw signal; (**b**) filtered signal.

## Data Availability

Data is contained within the article.

## Appendix B. Field Testing the Antenna as a Component of a Ground-Penetrating Vital Sign Detector

To verify whether the proposed antenna can be used as a radar sensor to detect human respiration through building material, a preliminary experiment was conducted, as shown in Figure A3. A simple bridge was constructed with nine wooden pallets as the supporting structure and 6 cm thick bricks as the top layer. A test person lay underneath the bridge. Two of the proposed antennas, one as the transmitting (Tx) antenna and one as the receiving (Rx) antenna, were placed on the top of the bricks at a distance of 50 cm. The radar sampling frequency in this experiment was 100 Hz.

The received raw signal was the transmission coefficient of $S_{21}$ over time. It contained a static offset and white noise due to the environment and linear drift due to the non-linear hardware components. Therefore, the signal should be firstly digitally filtered before further signal processing. In Figure A4a, the complex-valued signal of one 30 s measurement is presented with the real part and the imaginary part. A digital filter with two stages was then applied: In the first stage, a first-order Butterworth infinite-impulse-response (IIR) high-pass filter with a cutoff frequency of 0.06 Hz was used to remove the static offset and linear drift; in the second stage, a seventh-order Butterworth IIR low-pass filter with a cutoff frequency of 1.1 Hz was used to remove the noise. The periodic breathing pattern of the test person can be clearly identified from Figure A4b.
